# Pericardial cyst with right ventricular compression

**Published:** 2012-07-03

**Authors:** Julius Chacha Mwita, Peter Chipeta, Reuben Mutagaywa, Belson Rugwizangoga, Elijah Ussiri

**Affiliations:** 1 Muhimbili University of Health and Allied Sciences, Tanzania; 2 Muhimbili National Hospital, Tanzania

**Keywords:** Pericardial cysts, mediastinal tumors, ventricule, Tanzania

## Abstract

Pericardial cysts are infrequent and benign mediastinal lesions. While most pericardial cysts are asymptomatic, some patients may present with compression symptoms. We present the case of a 22-year-old man who presented with a right pericardial cyst that caused compression of the right ventricle.

## Introduction

Pericardial cysts are rare benign intrathoracic lesions that are frequently discovered incidentally in asymptomatic patients. They can also be symptomatic depending on their sizes and position. Symptoms may be secondary to cyst rupture, haemorrhage or its mass effect on bordering structures. We describe the case of a patient who had pericardial cyst compression of the right ventricle and who clinically presented with right heart failure.

## Patient and case presentation

A 22-year-old man was referred to our hospital because of lower limb swelling, abdominal distension that had progressed over the preceding 8 months. Prominent lower limb vessel distension and scrotal swelling were present during this time. He had no history of fever chest pain or cough.

The patient was clinically stable. He had bilateral pitting pedal edema, superficial lower limb venous distension and left varicocele. The blood pressure was 110/70 mm Hg and the heart rate was 82 beats/min and regular. The jugular venous pressure was elevated and a grade 2/6 systolic ejection murmur was found. The lungs were clear. Abdominal examination revealed hepatomegaly and ascites. A clinical diagnosis of right sided heart failure was made.

The electrocardiography detected sinus tachycardia of 104 beats per minutes, right axis deviation, an incomplete right bundle-branch block and no conduction disturbances. A chest X ray was normal. Ascites and hepatic congestion were revealed by an abdominal ultra- sonogram.

**Figure 1:**
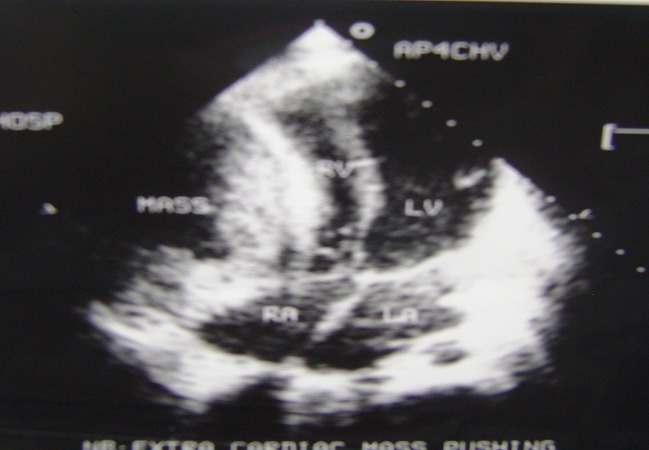
Transthoracic echocardiography showing an echolucent mass compressing the right ventricle

**Figure 2:**
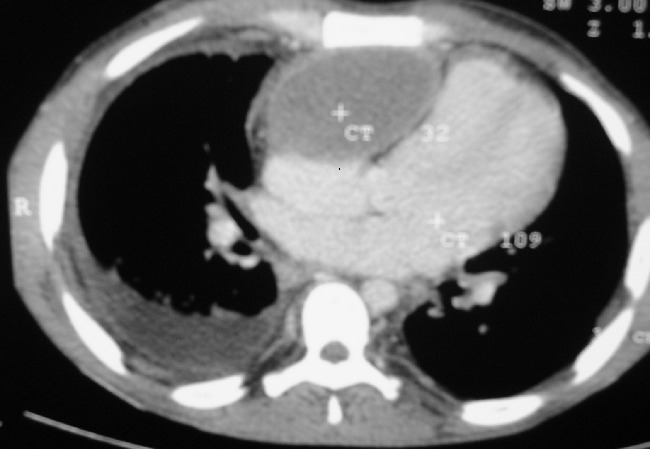
Axial CT image showing a retrosternal, anterior mediastinal fluid filled mass compressing the right ventricle and atrium. The mass has a well defined margin, non contrast enhancing and pushes RV to the left

A two dimensional transthoracic echocardiography showed an abnormal anterior mass that compressed the right ventricle and atrium (Figure 1). The mass was extrinsic and confined to the pericardium. The inferior vena cava and hepatic veins were dilated without respiratory variation in size of the inferior vena cava. Both left and right ventricular systolic functions were normal and there was no evidence of pericardial effusion. A computer tomography examination of the chest (Figure 2) was performed and showed a retrosternal fluid filled structure that had a well defined margin causing a significant compression of the right ventricle. The mass didn′t show any signs of contrast medium uptake and had a clear cleavage with the right ventricle. The inferior vena cava was prominent and there were no evidence of lymphadenopathy.

The patient was referred for a surgical removal of the cyst. A 15 × 10 cm anterior mediastinal cyst filled with necrotic debris was found and removed. It had thick walls and compressed both the right ventricle and atrium. Histology revealed a fibrovascular cyst wall with evidence of chronic inflammation and extensive necrosis. The patient had an unremarkable postoperative recovery and was discharged 10 days later. A follow-up transthoracic echocardiogram did not demonstrate any mass in the pericardium.

## Discussion

Pericardial cysts are infrequent lesions, accounting for about 7% of the mediastinal tumors [1]. They are found in one person per 100,000 [1]. While pericardial cysts can originate from every location, the left (51% to 70%) and right (28% to 38%) cardiophrenic angles represent the most common positions [2]. Histologically these cysts are lined with a single layer of mesothelial or endothelial cells, with the remainder of the wall composed of connective tissue with collagen and elastic fibers [3]. They have clear fluid-filled centre which distinguishes them from other pericardial masses. Presence of infection or hemorrhage may nevertheless alter the fluidity of the cysts [3].

Most patients are asymptomatic or have variable symptomatology, depending on the dimension, site of the cyst and associated complications. They are, for that reason, incidental diagnoses in asymptomatic patients. The most common symptoms are atypical chest pain, dyspnea, and persistent cough [4]. Reported complications include compression of adjacent tissues, spontaneous cyst rupture, hemorrhage into and infection [5]. Differential diagnoses of pericardial cysts include prominent pericardial fat pad, ventricular aneurysm, pericardial hematoma, and mediastinal tumours [6] Symptoms, in this case, were particularly related to the size of pericardial cyst, which compressed the right ventricle of the patient, causing hemodynamic alteration.

Pericardial cysts may be detected on radiographic examinations and diagnosed on echocardiography, computerized tomography, and nuclear magnetic resonance imaging. Echocardiographically a pericardial cyst appears as a homogeneous echolucent mass with an echo-free space indicative of its separation from the cardiac chambers [7]. Computer tomography or magnetic resonance imaging can be used to confirm the echocardiographic findings. Differentiating malignancies from non-malignant fluid-filled cysts may nevertheless be difficult.

Asymptomatic patients with pericardial cysts generally do not require any surgical intervention but rather a conservative treatment and close follow up depending on the location and size of the cyst. Symptomatic patients are typically managed by surgical removal of the cysts. Percutaneous aspiration of cyst contents can also be done as an alternative to surgical resection in symptomatic patients. Recurrences have not been reported after excision [2].

In this study, symptoms of right ventricular failure were present secondary to compression of the right chambers, as evidenced by echocardiography. Because of these symptoms, our patient underwent cyst resection. The perioperative period was uneventful and the patient has been in his in excellent clinical condition post operatively.

## Conclusion

This case demonstrates a rare differential diagnosis of right heart failure that can be treated surgically with complete improvement.
